# Automatic Detection for Multi-Labeled Cardiac Arrhythmia Based on Frame Blocking Preprocessing and Residual Networks

**DOI:** 10.3389/fcvm.2021.616585

**Published:** 2021-03-19

**Authors:** Zicong Li, Henggui Zhang

**Affiliations:** ^1^Biological Physics Group, Department of Physics and Astronomy, The University of Manchester, Manchester, United Kingdom; ^2^Peng Cheng Laboratory, Shenzhen, China; ^3^Key Laboratory of Medical Electrophysiology of Ministry of Education and Medical Electrophysiological Key Laboratory of Sichuan Province, Institute of Cardiovascular Research, Southwest Medical University, Luzhou, China

**Keywords:** electrocardiogram, cardiac arrhythmia, residual neural network, attention-based bidirectional, long short-term memory, frame blocking, auto-detection algorithm

## Abstract

**Introduction:** Electrocardiograms (ECG) provide information about the electrical activity of the heart, which is useful for diagnosing abnormal cardiac functions such as arrhythmias. Recently, several algorithms based on advanced structures of neural networks have been proposed for auto-detecting cardiac arrhythmias, but their performance still needs to be further improved. This study aimed to develop an auto-detection algorithm, which extracts valid features from 12-lead ECG for classifying multiple types of cardiac states.

**Method:** The proposed algorithm consists of the following components: (i) a preprocessing component that utilizes the frame blocking method to split an ECG recording into frames with a uniform length for all considered ECG recordings; and (ii) a binary classifier based on ResNet, which is combined with the attention-based bidirectional long-short term memory model.

**Result:** The developed algorithm was trained and tested on ECG data of nine types of cardiac states, fulfilling a task of multi-label classification. It achieved an averaged F1-score and area under the curve at 0.908 and 0.974, respectively.

**Conclusion:** The frame blocking and bidirectional long-short term memory model represented an improved algorithm compared with others in the literature for auto-detecting and classifying multi-types of cardiac abnormalities.

## Introduction

Cardiac arrhythmias refer to irregular heart rhythms, representing abnormal cardiac electrical activities associated with abnormal initiation and conduction of excitation waves in the heart (1). Cardiovascular diseases in association with cardiac arrhythmias can cause heart failure, stroke, or sudden cardiac death (2). Early detection and risk stratification of cardiac arrhythmias are crucial for averting severe cardiac consequences. With their ability to represent useful information regarding the electrical activity of the heart, electrocardiograms (ECG) measured via electrodes placed on the body surface played an important role in diagnosing cardiac abnormalities (3). Recently, artificial intelligence-based algorithms (4, 5) have shown promises in screening abnormal features of ECG to achieve an automatic diagnosis of cardiac arrhythmias with high accuracy but less labor demand.

In previous studies, several auto-detection algorithms have been developed (6, 7). These algorithms focus on extracting physiological features of ECGs, such as heart rate variation (calculated from the time interval between two consecutive R peaks), the width of the QRS complex, and QT intervals. However, these algorithms do have limitations for practical application, as ECG features were merely extracted from RR or QT intervals, providing insufficient information for multiple types of cardiac event classification. To extract sufficient features automatically and achieve high classification accuracy, recent advancements in deep neural network (8) helped to develop several improved auto-detection algorithms (5, 9, 10) for ECG analysis and classification. These studies illustrated that the deep-learning-based algorithms have the advantages of extracting and processing ECG features automatically.

However, the algorithms discussed earlier are mainly focused on processing single-lead ECG rather than the 12-lead ECG, which is commonly used in the clinical setting for providing more diagnostic information than a single-lead ECG on cardiac excitations (11). Also, it is still a challenge to auto-detect multi-types of cardiac diseases based on 12-lead ECG due to (i) similar morphological features of ECG among different types of diseases, such as between atrial fibrillation (AF) and premature atrial contraction (12); (ii) imbalanced ECG data for various heart diseases in some training datasets, which may result in excessive bias or over-fitting of the neural network for diagnosis; (iii) unequal recording length of clinical ECG recordings, which may result in loss of some essential signals in the process of preprocessing for training the neural network.

Therefore, this study aims to develop a novel method for preprocessing raw ECGs and design an appropriate neural network for classifying 12-lead ECG data with multi-labeling and varied lengths.

## Methodology

**Figure 1:**
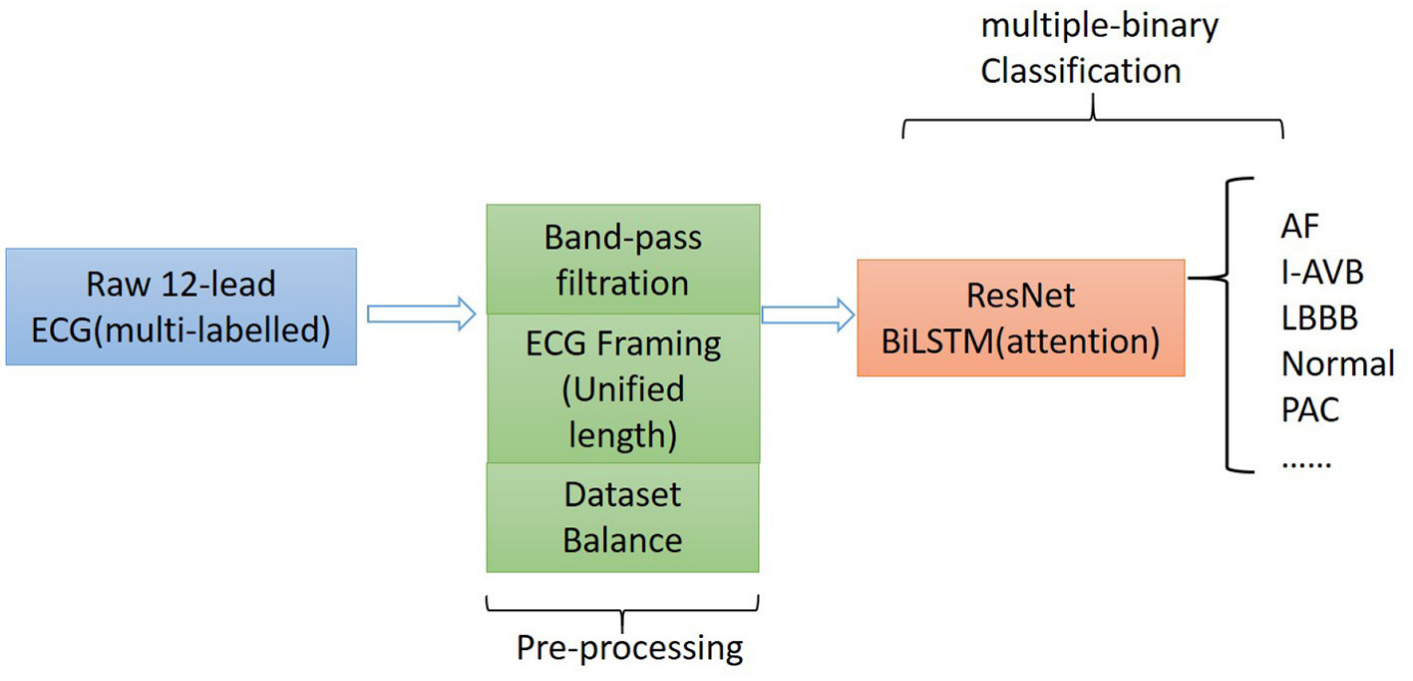
Flow chart diagram of the algorithm for multi-type cardiac arrhythmia classification.

The proposed algorithm for classifying 12-lead ECG with multi-labeling consists of components of data denoising, framing blocking, and dataset balance for data preprocessing and a neural network structure based on ResNet in combination with attention-based bidirectional long short-term memory (BiLSTM). The general structure of the proposed algorithm is shown in Figure 1.

### Dataset Description

#### China Physiological Signal Challenge in 2018

The China Physiological Signal Challenge (CPSC) 2018 dataset consists of 6,877 (females: 3,178; males: 3,699) recordings of 12-lead ECG data collected from 11 hospitals. Each recording is saved as a MAT file with a hea file presenting labels and relevant information of the ECG recording at the end of the file. The ECG recordings are sampled at 500 Hz with different recording lengths, ranging from 6 to 60 s. The dataset contains ECG recordings for nine types of cardiac states, including AF, intrinsic paroxysmal atrioventricular block, left bundle branch block (LBBB), normal heartbeat (Normal), premature atrial contraction (PAC), premature ventricular contraction (PVC), right bundle branch block (RBBB), ST-segment depression (STD), and ST-segment elevation (STE). To illustrate the morphological variation of the ECG among different cardiac states, the visualization of ECG lead II waveforms for nine types of cardiac states and a multi-labeled ECG recording can be found in Supplementary Figures 1, 2, respectively. Among the 6,877 recordings, 476 of them have two or three different labels. Table 1 lists the numbers and distribution of eight-type cardiac arrhythmias in the 476 multi-labeled recordings of the CPSC 2018 dataset.

**Table 1:** Numbers and distribution of ECG recordings with multiple labels (13) for eight different types of abnormalities in CPSC 2018.

	**AF**	**I-AVB**	**LBBB**	**RBBB**	**PAC**	**PVC**	**STD**	**STE**
AF	0	0	29	172	4	8	33	2
I-AVB		0	8	10	3	5	6	4
LBBB			0	0	10	6	3	4
RBBB				0	55	51	20	19
PAC					2	3	6	5
PVC						0	18	2
STD							0	2
STE								0

#### China Physiological Signal Challenge in 2020

An independent dataset, the CPSC 2020 dataset, is also used for testing the robustness of the proposed model. The dataset from CPSC 2020 contains two subsets of annotated recordings, one with 6,877 (males: 3,699; females: 3,178) recordings and the other with 3,453 (males: 3,453, females: 1,610) recordings of 12-lead ECG data, each of which was collected by a sampling frequency of 500 Hz. Furthermore, the dataset from CPSC 2020 contains public and unused datasets from the CPSC 2018 dataset for seven common types of cardiac states, details of which are listed in Table 2 for the total number and distribution of cardiac abnormality in the CPSC 2020 dataset. Except for normal heart rhythm, the numbers and distribution of six types of abnormalities in multi-labeled recordings of the CPSC 2020 dataset can be found in Supplementary Table 1. In the experimental process, the total recordings for seven common types of cardiac states in CPSC 2020 were used for robustness testing.

**Table 2:** Recording numbers and distribution of seven types of abnormalities in CPSC 2020.

**Abnormalities**	**CPCS 2020**	**CPCS 2020**	**Total**
	Training	Training	
	set1	set2	
AF	1,221	153	1,374
I-AVB	722	106	828
LBBB	236	38	274
Normal	918	4	922
RBBB	1,857	1	1,859
PAC	616	73	689
PVC	0	188	188

#### PTB XL

To demonstrate the universality and robustness of the proposed algorithm, the cross-validation of the algorithm was processed on the PTB XL dataset. The PTB XL dataset comprises 21,837 clinical 12-lead ECG records from 18,885 patients (males: 9,820, females: 9,064) of 10-s length. As a multi-labeled dataset, the ECG records were annotated by two cardiologists based on the Standard Communication Protocol for Computer-Assisted Electrocardiography standard (14). Table 3 illustrates the distribution of diagnosis, where the diagnostic labels are aggregated into superclasses.

**Table 3:** Recording numbers of distribution of five types of diagnostic labels in PTB XL.

**Superclass**	**Description**	**Record_Num**
NORM	Normal ECG	9,528
MI	Myocardial Infarction	5,486
STTC	ST/T Change	5,250
CD	Conduction Disturbance	4,907
HYP	Hypertrophy	2,655

### Preprocessing

#### Noise Processing

Most ECG signals have a frequency range between 0.1 and 35 Hz and are non-stationary in the low-frequency range (15). Noises normally contaminate them from sources of power-line interference, muscle movement, and baseline wander, which blur the features of the ECG signals for classification. For minimizing possible effects of noise on model classification, raw ECG data in the two databases were denoised by using an eight-order Butterworth lowpass (35 Hz) filter for eliminating noise and removing baseline wander.

#### Frame Blocking

**Figure 2:**
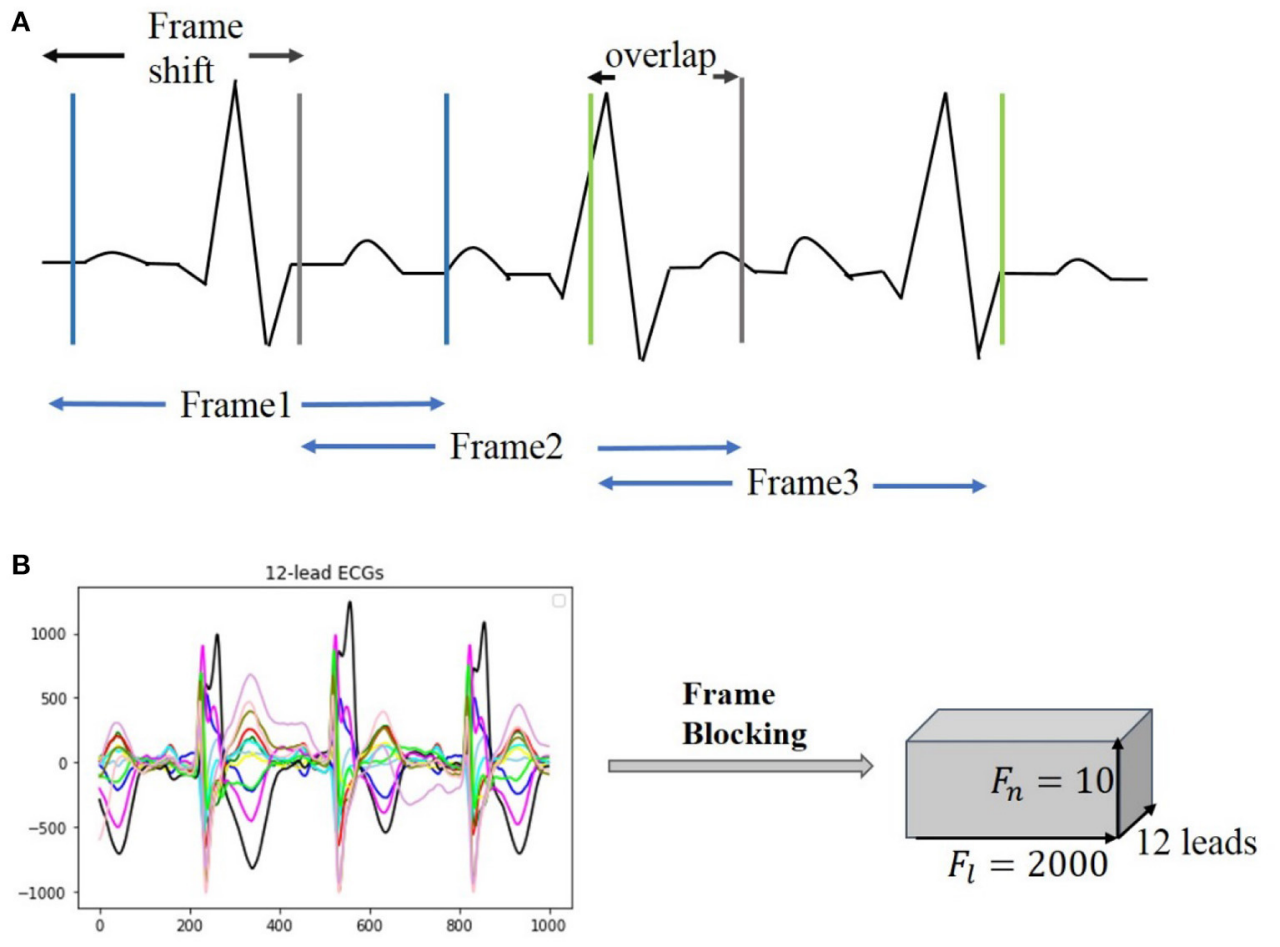
Illustration of frame blocking for pre-processing ECG signal. **(A)** Method of frame blocking. **(B)** Example of 12-lead ECG data segments after frame blocking processing.

Clinical ECG data are normally collected with non-uniform duration, ranging from 10 s to 24 h, causing difficulties for training and testing neural networks. For unifying the length of each of the ECG recordings, a frame blocking method adapted from speech recognition (16) is utilized in the present study. In speech recognition, frame blocking is used to segment speech signals into short frames with overlapping, enabling a smooth transition between adjacent frames that maintains the continuity of the signal. As there is a similarity between speech signals and ECG time series (17, 18), the frame blocking method can be implemented in ECG data for unifying their recording length. Figure 2A illustrates the implementation of the frame blocking method on the cardiac signal. In the figure, *F*_*s*_, the frameshift, denotes the time lag of the frame (from the starting time of the ECG recording), and *f*_*o*_ denotes the overlapping part between adjacent frames. Thus, the length of each frame, *F*_*l*_, can be expressed as:

(1) $$\begin{array}{r} F_{l}=F_{s}+f_{o} \end{array}$$

For a raw ECG recording with a total length of *S*_*l*_, given the number of frames *F*_*n*_ and frame length *F*_*l*_, then the framing equation can be represented as:

(2) $$\begin{array}{r} F_{s}=\left(S_{l}-F_{l}\right)/\left(F_{n}-1\right) \end{array}$$

The length of each ECG recording in the CPSC 2018 dataset is variable, of which 6,634 recordings have their length shorter than 40 s (i.e., ~20,000 sampling data points). To retain the available ECG signals for each record as much as possible, we set *F*_*l*_ and *F*_*n*_ as a constant of 2,000 (sampling points) and 10, respectively, but *F*_*s*_ variable for fitting the required length and number of frames. Figure 2B illustrates an example of a 12-lead ECG recording processed by the frame blocking, with each ECG recording can be transformed into a frame-block with a uniform size [i.e., (*F*_*n*_, *F*_*l*_, lead_num)]. As such, the frame blocking acted on each lead of the signals and divided them into 10 frames with a frame length of 2,000 sampling points.

#### Dataset Balance

In the present study, the multi-labeled dataset was converted into multiple types of sub-dataset classes, each of which represented one of the multiple types of cardiac states. The length of the ECG data for each type of cardiac abnormalities is imbalanced, leading to over-fitting and weak generalization of the proposed neural network. To address this problem, a random under-sampling method (19) is used. For training and testing each binary-classifier, data samples are selected randomly from the dataset until a 2:1 ratio of samples in the majority class to the minority class is obtained.

### Construction of the Model

#### Residual Convolution Neural Network

**Figure 3:**
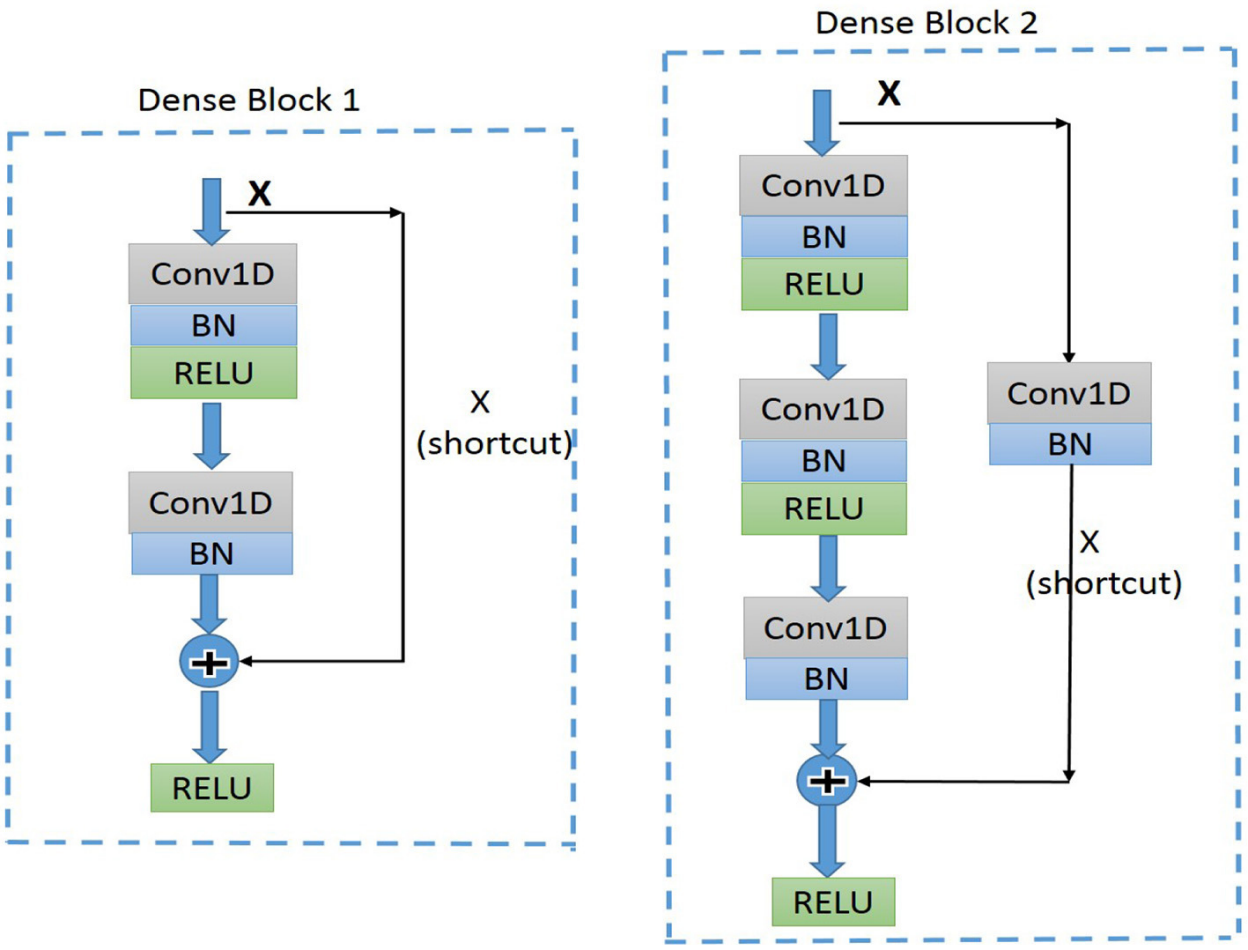
Diagram of the structure of dense block1 and dense block2. BN, batch normalization; ReLU, rectified linear units; Conv1D, one-dimension convolutional layer.

Residual convolutional neural network (CNN) (20) has shown excellent performance on image recognition for addressing the degradation problem of a deeper neural network, and it is believed to be useful for analyzing time-series signals, such as ECG. Here, we implemented one-dimension residual CNN with 13 layers based on the structure of ResNet. As shown in Figure 3 for the general structure of the network, both dense blocks 1 and 2 belong to the residual block, and the shortcut connection simplifies the optimization of the deep neural network.

#### Attention-Based Bidirectional Long Short-Term Memory

**Figure 4:**
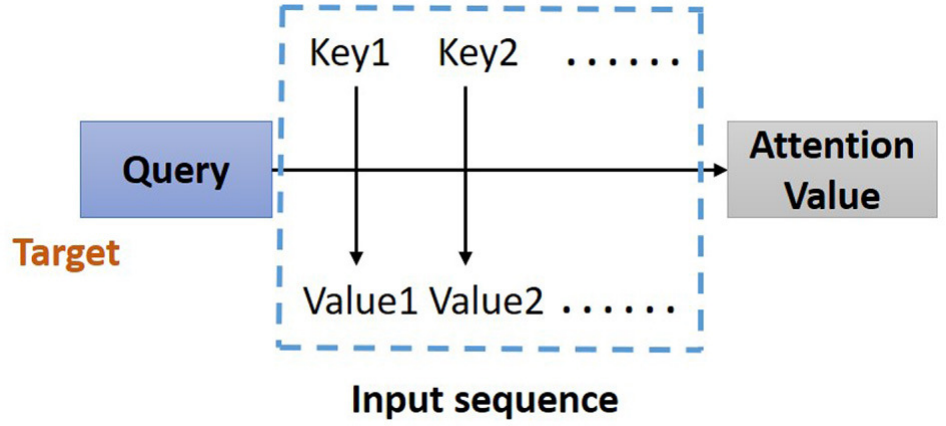
Structure of Attention Mechanism. Correlation between the key-value pairs of the input time sequences and the query (a condition value) is evaluated, based on which the weight of each value is calculated. Through the weighted summation, the attention value for each element in the input time sequence can be assign.

In the proposed model, the residual blocks primarily focus on extracting features from ECG signals, and the attention-based BiLSTM structure focuses on learning and analyzing the feature map produced by the residual blocks. The bidirectional structure provides contextual information in the forward and backward directions for the output layer, providing more prediction information (21); thus, in this study, a BiLSTM (17) is used to catch some essential information from a long-distance correlation of the ECG data. The proposed model implements the Attention Mechanism (Figure 4) to allocate different attention values to each input query, which assists BiLSTM to precisely identify valid information and reduce the loss of key features. The attention-based BiLSTM can focus on the essential part of the input, meanwhile, it catches global, and local connection precisely because of the weight and attention allocation for the input time sequences.

#### Structure of the Proposed Network

**Figure 5:**
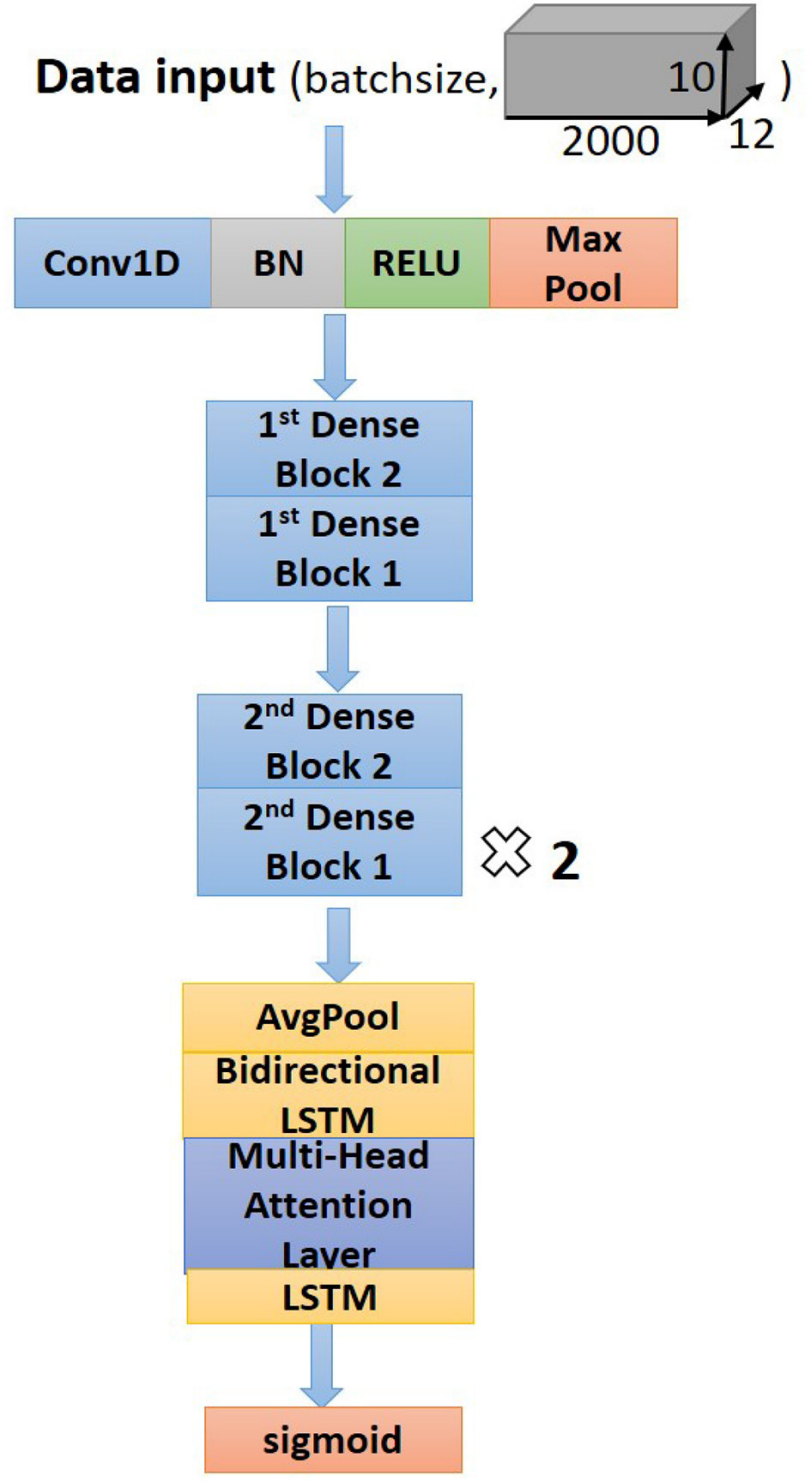
Structure of the proposed neural network.

The dense block 1 shown in Figure 3 is a standard residual block in ResNet. It consists of two one-dimension convolutional layers (Conv1Ds), two Batch Normalization (BN) (22), and rectified linear units (ReLU) (23) for the activation function layers, as well as a shortcut connection that transmits the input to output directly before applying the second ReLU nonlinearity. As for the structure of dense block 2, a Conv1D layer and BN are added in a shortcut for adjusting channels or stride to fit the desired shape of output. The overall structure of the proposed model is shown in Figure 5.

Following the Conv1Ds are the BN and ReLU layers, which help to simplify the parameter adjustment, improve the learning speed, and address the vanishing gradient problem of the model. Then a 1D max-pooling layer is used to down-sample the feature map by computing and extracting maximums of every three values in the feature map matrix, thus retaining the most valuable features and avoiding unnecessary memory usage during the training process. After the max-pooling layer, dense block 2 is connected to dense block 1 to fulfill a complete residual CNN. Before processing the attention-based BiLSTM for feature analysis, the global average pooling layer (24) is used to process the regularization of the global structure of the network, preventing it from overfitting.

Supplementary Table 3 lists a set of optimal parameters for each layer and the residual blocks. Among different residual blocks in different positions (first or second), convolution kernels have different sizes and numbers. For classification, sigmoid activation with binary cross-entropy (25) is used to convert the output sequence from the last LSTM layer into a probability for a specific label, based on which classification is determined with a given threshold.

### Experimentation Details and Evaluation Matrix

The proposed model is initially trained and implemented using the CPSC 2018 datasets and run on Tesla T4 GPU with Keras frameworks (26). As described in the section of dataset balance, the positive and negative samples of each cardiac abnormality with the ratio of 1:2 are randomly selected and combined as input datasets for the model. For each binary classifier, the input data were divided into three subsets: 64% for training, 16% for validation, and 20% for testing. The 5-fold cross-validation was also implemented for training and validation. The test dataset was used purely for evaluating the performance of the model and was not involved in training and validation of the proposed model.

The classification performance can be comprehensively evaluated by precision, Recall, F score, receiver operator characteristic (ROC) curve, and area under the curve (AUC). These evaluated measures are calculated by the following equations:

$$\begin{array}{l} Precision_{i}=\frac{TP_{i}}{TP_{i}+FP_{i}}\\ Recall_{i}=\frac{TP_{i}}{TP_{i}+FN_{i}}\\ F1score_{i}=\frac{2\times \left(Precision_{i}\times Recall_{i}\right)}{Recall_{i}+Precision_{i}} \end{array}$$

In these equations, *i* denotes each of the types of cardiac arrhythmias. *TP*_*i*_ and *TN*_*i*_ represent the number of correctly predicted positive and negative samples, respectively. On the other hand, *FP* and *FN* are the values of false prediction for positive and negative samples separately. The ROC curve measures the performance of the model *via* plotting the trade-off between sensitivity and specificity, and the AUC is the value of the area under the ROC curve. A ROC curve is closed to the top-left corner and has the AUC close to 1 indicates the good performance of the classification model.

## Result

### Adjustment of Hyperparameter

The process and outcome of tunning of hyperparameters can be found in the Supplementary Material.

### Comparison of Model Performance to Different Model Structures

To compare the performance of the proposed model to others, results obtained here were compared with those obtained from multiple models with different network structures, which included (i) the plain CNN with attention-based BiLSTM; (ii) Plain CNN + LSTM; and (iii) Challenge-best deep neural network model.

i) Plain CNN + attention based BiLSTM

Supplementary Table 5 lists the architecture of plain CNNs and attention-based BiLSTM. Except for the structure of shortcut, the convolutional layers, batch normalization layers, and ReLU layers of this model are similar to those of the proposed model. Multiple dropout layers were added to this structure, which could reduce the complexity of coadaptation between hidden neurons and improve the robustness of the neural network (27).

ii) Plain CNN + LSTM

Similar to the plain CNN + attention-based BiLSTM model, the structure of the plain CNN + LSTM model contains plain CNNs without shortcut. Moreover, the attention-based BiLSTM is replaced by LSTM layers with a simpler structure for feature analysis.

iii) Challenge-best deep neural network model

Supplementary Table 6 depicts the structure of the first prize model (13) for the automatic diagnosis of cardiac abnormalities in the CPSC 2018 dataset. The model consists of five CNN blocks and attention-based bidirectional GRU. Each block includes two convolutional layers, with one pooling layer appended for reducing the over-fitting and the amount of computation. To achieve optimal performance of classification, the bidirectional GRU layer followed by an attention layer is connected to the last convolutional block. Moreover, the hyperparameters of the challenge-best model have been modified based on our proposed model, enabling a direct comparison.

**Figure 6:**
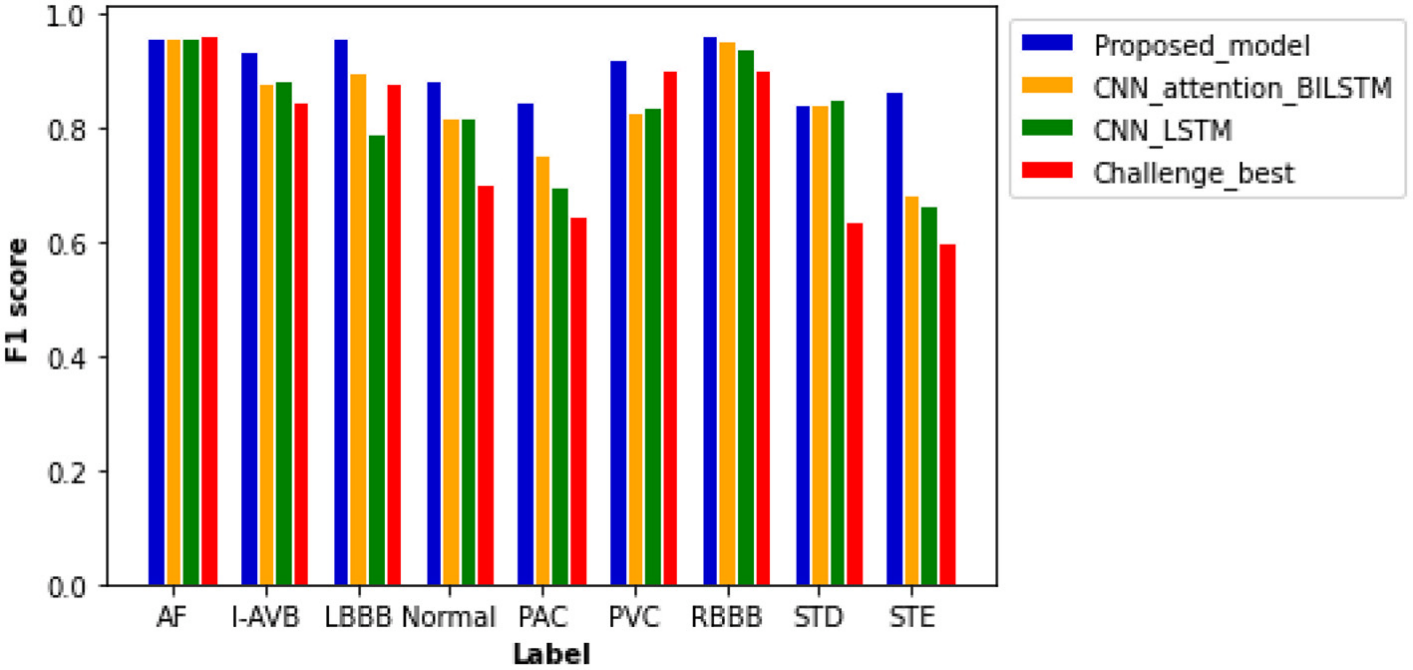
Comparison of F1 scores between different models based on the same test samples. F1 scores of the proposed model show the best performance of the model as compared with others with values of 0.959 for AF, 0.937 for an intrinsic paroxysmal atrioventricular block (I-AVB), 0.958 for LBBB, 0.885 for Normal, 0.848 for PAC, 0.920 for PVC, 0.965 for RBBB, 0.841 for STD, and 0.868 for STE. Specific values of F1 scores of the other three models are shown in Supplementary Table 7.

Figure 6 plots computed F1 scores achieved by the proposed model, which are compared with results from other comparable models using the same dataset. As shown in the figure, the F scores of six labels in the proposed model are notably higher than others. The proposed model achieved the highest F score of 0.965 for the RBBB case, followed by 0.959 and 0.958 for AF and LBBB, respectively. The probability results illustrated by the confusion matrix (Supplementary Figure 3) demonstrated a low probability of misclassification by our proposed model; especially, the probability of false positive and false negative for AF, LBBB, PVC, and RBBB is closed to zero.

The plain CNN with attention-based BiLSTM ranks second with an average F score of 0.846. The computed F scores from the model for PAC, PVC, and STE are much smaller than those of the proposed model. Thus, the replacement of residual networks reduced the performance of the model. The performance of plain CNN with the LSTM model is not optimal for each type of cardiac abnormalities, especially for the cases of LBBB, PAC, and STE, for which F score is <0.800. Although the Challenge-best model achieved the highest F score for the AF case, its performance for other abnormalities is relatively poor. Over-fitting occurred when the Challenge-best model was implemented for the data input of PAC, STD, and STE, leading to undesired F scores. Though the architecture and hyperparameters of the Challenge-best model are similar to the model shown in Supplementary Table 5, the computed average F score of the challenge-best model is much lower as compared with the presented model.

Supplementary Figure 4 shows the computed ROC curve from different models for each type of the nine cardiac states. Comparing with other models, the ROC curve of the proposed model is closer to the top left corner, with an averaged AUC at 0.974, suggesting out-performance to the other models.

### Performance on Different Preprocessing

To illustrate the advantage of the frame blocking for pretreatment of the data, the performance of the proposed model was compared with that using a common preprocessing method (28–30), which uses direct cutting and zero-padding protocol to unify the length of ECG signals. As for a fixed length of 40-s ECG data (i.e., 20,000 sampling data points), the common method can either truncate the exceeding signal samples when the length of original records exceeds 40 s or pads zeros to the data when the length is <40 s.

**Figure 7:**
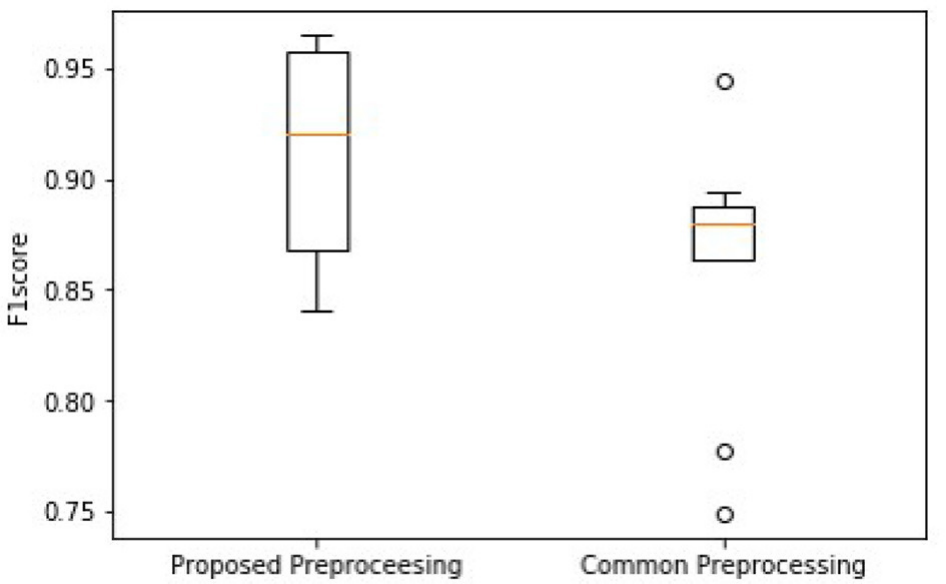
Comparison of overall F1 scores between using the proposed block framing and the common padding method for pre-processing ECG data for classification.

As shown in Figure 7, the higher median and minimum of the common method illustrate an improved model performance of the proposed frame blocking method. Moreover, the distribution of F1 scores by the common method is discrete, reflecting the instability of the performance of the classification model. To further evaluate the significant difference of this observation, the Wilcoxon signed-rank test is done on the two paired of F1scores. The *p-*value is 0.028 (<0.05), revealing the difference between F1scores produced by two pretreatments is significant.

### Robustness Testing

**Figure 8:**
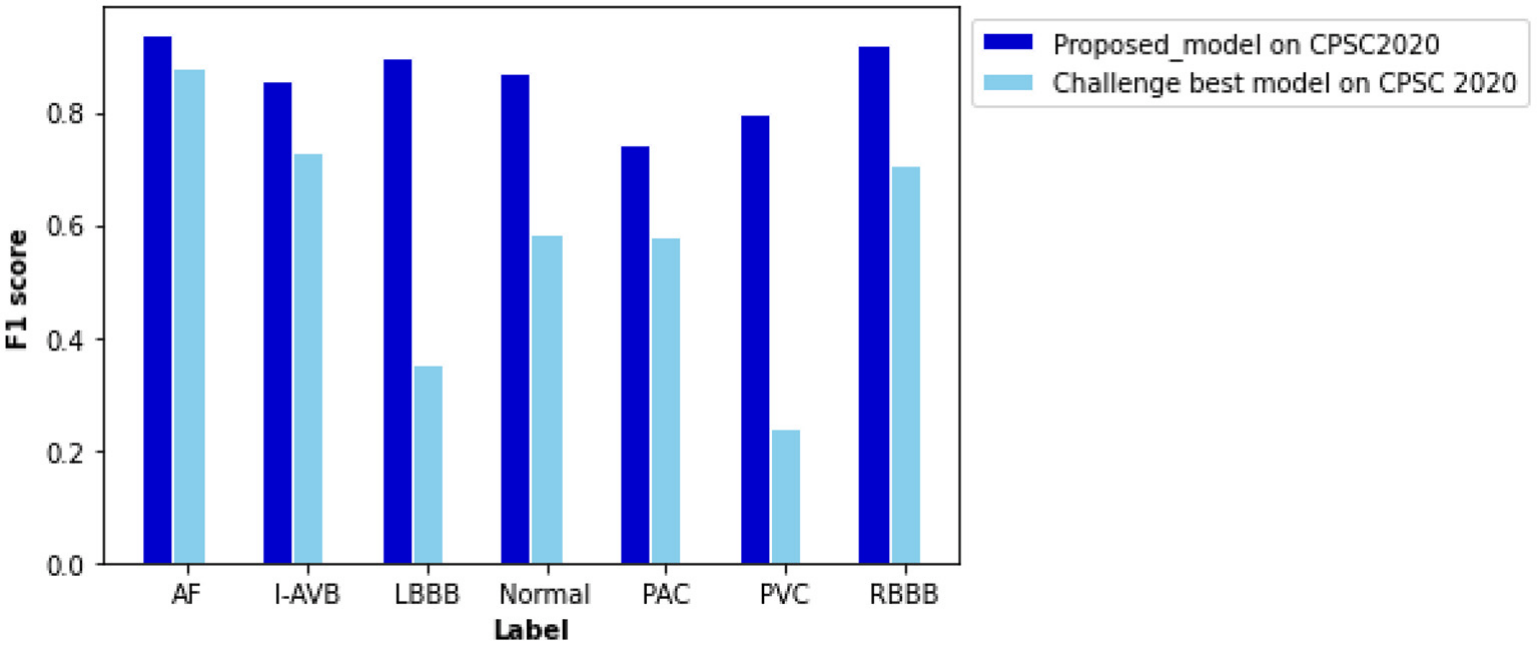
Comparison of performance between the proposed model and the Challenge-best model tested on the CPSC 2020 dataset for various types of arrhythmias. F scores of the proposed model are 0.940 for AF, 0.856 for intrinsic paroxysmal atrioventricular block (I-AVB), 0.898 for LBBB, 0.870 for Normal, 0.743 for PAC, 0.798 for PVC, 0.922 for RBBB, 0.841 for STD, and 0.868 for STE. Comparison of the F1 score between them is listed in Supplementary Table 8.

Being tested on the CPSC 2020 dataset, the proposed model shows F1 scores significantly higher than those of the Challenge-best model (13) for all seven types of cardiac arrhythmias (Figure 8). The computed ROC curve and AUC (shown in Supplementary Figure 5) also demonstrate the better performance of the proposed model (with an averaged AUC of 0.951) than the challenge-best model (13). It is interesting to note that the Challenge-best model is much harder to converge on the CPCS 2020 than those of CPSC 2018. Also, the performance of the challenge-best model varies dramatically for different types of cardiac abnormalities with the use of the CPSC 2020 dataset as indicated by low values of F1 score for LBBB, Normal, PAC, and PVC conditions.

### Cross-Validation

**Figure 9:**
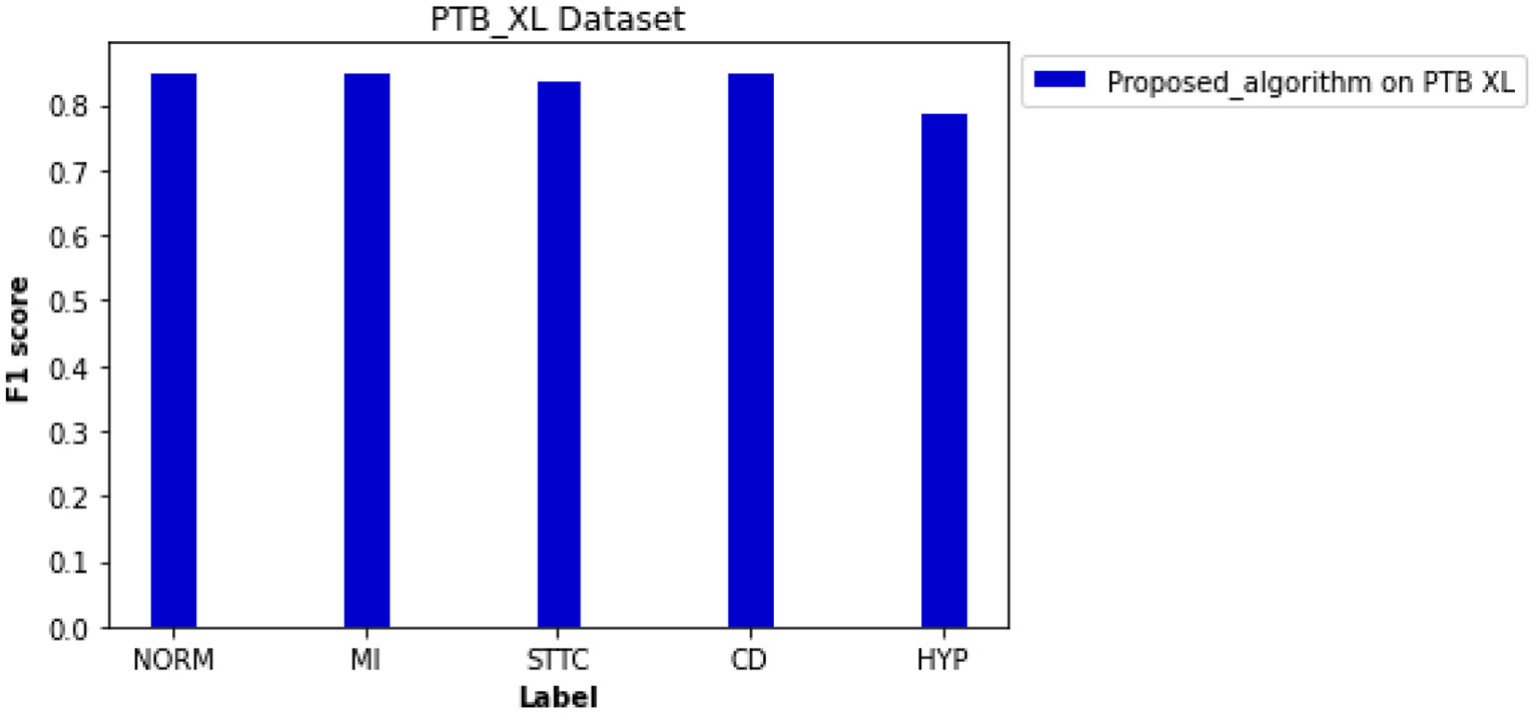
Performance of the proposed algorithm on PTB XL dataset for five diagnosis labels. F scores of each label are 0.853 for NORM, 0.852 for MI, 0.842 for STTC, 0.853 for CD, and 0.791 for HYP.

Besides the CPSC datasets, the PTB XL dataset was adapted for cross-validation of the proposed novel algorithm for preprocessing and classification. As shown in Figure 9, the F1 scores of four diagnosis labels are higher than 0.800, achieving an average F1 score of 0.838 for all diagnosis labels in that dataset. The computed ROC curve and AUC (shown in Supplementary Figure 6) also illustrated a satisfying performance of the proposed algorithm on an external dataset with an average AUC of 0.950.

## Discussion

The novelty and major contributions of the present study are the following: (i) we proposed a preprocessing algorithm of frame blocking adapted from speech recognition, which decomposes ECG signals into overlapped frames. The proposed frame blocking method minimizes the loss of valid signals while maintaining the continuity of ECG signals in the process of unifying the length of variant ECG recordings; (ii) we developed a neural network based on the residual networks (31) with attention-based BiLSTM. As compared with the previous algorithms mentioned earlier for ECG detection, the presented network can extract and analyze ECG features automatically, thereby improving the model performance. It also alleviates the vanishing and exploding gradient problem as seen in deep neural networks, and (iii) by training and testing the model using three independent datasets of 12-lead ECG signals provided in CPSC (32) and PTB XL (33), the proposed algorithm demonstrates superiority and robustness in classifying 12-lead ECGs with multi-labeling.

In recent years, numerous automatic detection methods for ECG analysis and classification have been developed. These methods are mainly based on and tested using the open-source MIT-BIH database (34), which are mainly single lead ECGs with single labeling. Thus, the general applicability of these algorithms for automatic stratifying multi-leads ECG and multiple types of arrhythmias is unclear. In this study, we developed a new algorithm based on frame blocking and the structure of ResNet, in combination with attention-based BiLSTM. Initially, the novel algorithm was trained and evaluated on the datasets of CSPC for classifying 12-lead ECG for nine types of arrhythmia labeling. By comparing the performance of other model structures (Figure 6), the superiority of the proposed model was confirmed. Comparing with the common preprocessing method (Figure 7), the frame blocking method reduces the number of zeroes padded at the end of the signal recording, enhancing the valid part of ECGs, as well as the autocorrelation of ECG records. Thus, the proposed preprocessing method is more conducive to feature extraction for further classification.

The proposed algorithm demonstrated its robustness and clinical value via robustness testing and cross-validation. Through the robustness testing, the proposed algorithm shows a consistent performance on the two datasets and various types of abnormalities, illustrating the robustness of the proposed algorithm and hyperparameters. Considering the cross-validation, both the frame blocking method and classification model are also applicable to the PTB XL dataset with a vast number of clinical records.

Regarding the model structure, the proposed model adopts a similar neural network structure as the Challenge-best model. Both are based on a bidirectional recurrent neural network with the attention mechanism, but the proposed model used residual networks to avoid gradient explosion and vanishing. The strength of ResNet has also been demonstrated by several studies (31, 35). In their studies, He et al. (35) showed the deep residual networks achieved an overall F1 score of 0.806. Rajpurkar et al. (31) utilized a 34-layer residual neural network to classify 65,000 multi-lead ECG records with 14 classes of cardiac disease and achieved an average accuracy and F1 score of 0.800 and 0.776, respectively. Due to the differences between the original dataset and preprocessing method, the crosswise comparison of classification models is not persuasive. The studies mentioned earlier processed the classification *via* complex network structures and a large amount of annotated data. Although the deeper neural networks with sufficient training data contributed high classification accuracy, the computation of the model also increased and required expensive hardware support. Our model adapted a similar structure as the studies mentioned earlier but simplified the network structure, raising the computational efficiency of training and the probability of clinical practice.

As for the challenge-best model proposed by Chen et al. (13), its whole structure contains 10 plain convolutional layers and 5 pooling layers. The use of unnecessary multiple layers in the CNN layers may reduce the model performance on a small and unbalanced dataset due to over-fitting, causing difficulties in parameter tuning. Thus, the occurring of the internal covariate shift slows down the training process when the input distribution changes, impairing the convergence ability of the model. Different preprocessing methods may also affect the performance of the model. We have looked at this issue. Compared with the commonly used method, the frame blocking method used in this study demonstrated its advantage in retaining maximum valid cardiac signal, which contributed to signal enhancement. Therefore, it proved to be a feasible preprocessing method to help the model extract more available features that are useful for model classification.

As for algorithms for multi-label classification (36), they can fall into problem transformation and algorithm adaption. With the development of neural networks, more studies (12, 31, 35, 37) designed an adaptive algorithm for multi-label classification. However, algorithm adaption has a high demand for sufficient training data and effective parameter adjustment to reduce misdiagnosis for multi-labeled ECG. Additionally, algorithm adaptation requires a complex model with proper parameters, increasing training cost and difficulties in data interpretation. In this study, each abnormality is considered as an independent binary problem, improving the interpretation of the features extracted. Although the binary relevance method cannot provide information about label correlation and interdependence directly, it still demonstrated some advantages for multi-label classifying performance and efficiency.

Regarding several recent studies (38–40), the risk stratification is in high demand to prevent sudden death or stroke caused by cardiac diseases. Inspired by the present algorithm, the risk prediction of cardiac diseases can be automated based on the clinical data collected from the ECG or electronic heart records. The shortcut connection in the residual network saved the computing time of the model and accelerated the convergence of the model, which is friendly to the clinical research setting. Thus, the model has the potential to automatically identify the patients at a high risk of cardiac diseases, process early clinical interventions and therapy. Furthermore, the application of a warning system of cardiac arrhythmias can be implemented based on the risk stratification and auto-detected algorithm. The ECG and electronic heart records can be stored and processed via cloud infrastructure and the internet, realizing the real-time monitoring system for cardiac arrhythmias and improving the early warning for the patients suffered from cardiac diseases.

## Related Works

Previous works into ECG auto-detection are mainly focused on manual feature extraction via the analysis in the time domain, frequency domain, and ECG morphology. After feature extraction, machine learning methods, such as Support Vector Machine (41) and linear discrimination analysis (42), are usually used for classifications. Compared with the algorithms mentioned earlier, ECG auto-detection based on deep neural networks focuses more on automatic feature extraction from ECG signals.

Hannun et al. (10) developed a deep CNN model for auto-detection of 12 classes of cardiac rhythms, achieving an averaged F1 score of 0.837. Besides, models based on LSTM have also been developed for processing ECG data with varied recording lengths and long-term time dependence to avoid the loss of valid features (43). For multiple label classification, the combined use of different neural networks demonstrates a better performance than the network structure purely based on the convolution layer. For example, the algorithm of multi-information fusion neural networks (44) consisting of BiLSTM and CNN has the advantages of simultaneously extracting the morphological features and temporal features, yielding an accuracy of 99.56%. Moreover, a similar BiLSTM–CNN model has been introduced to process data with long-term correlation, which could sufficiently extract features (45) to achieve high sensitivity and specificity of 98.98 and 96.95%, respectively.

The ECG auto-diagnosis algorithms discussed earlier demonstrated the advantages of deep learning algorithms in classification accuracy but were less focused on processing the 12-lead ECG with multiple diagnosis labels. Thus, it is in demand to develop an effective and auto-diagnostic algorithm to classify 12-lead ECG data for multiple cardiac arrhythmias.

## Limitation of Study

There are a few potential limitations in this study. Firstly, random under-sampling was used to address the imbalanced datasets of MIT-BIH (34), PTB XL (33), and CPSCs 2018 (32) and 2020. However, some potentially important and information-rich data might be discarded from the majority class, causing difficulties in fitting the decision boundary between majority and minority samples (19). Although the proposed model demonstrated good performance on two CPSC datasets (2018 and 2020) and PTB XL for 9, 7, or 5 different rhythmic abnormalities, it still needs to be further tested and improved by using other ECG datasets with more types of rhythmic abnormalities. However, as the types of rhythmic abnormalities increase, it would be expected that the required training time and GPU memory usage will be substantially increased.

In addition, the proposed neural network algorithm is heavily dependent on a large amount of annotated training data, which is labor expensive. For some rare types of cardiac abnormalities, it is difficult to collect such a large ECG dataset with annotation. In following-up works, it warrants to study further how algorithm adaption method (46) and other neural network architectures (47–49) help to deal with multi-labeled data directly and reduce time-demand for training. Moreover, unsupervised and semi-supervised learning can also be tested for addressing the lack of enough annotations.

## Conclusion

This study proposed a new framing preprocessing method that can minimize the loss of ECG signals to enhance the features of signals. The proposed algorithm can diagnose multiple types of cardiac arrhythmias with promising accuracy, clinical value, and robustness, which may be potentially useful in assisting risk stratification, clinical diagnosis, and real-time ECG monitoring. Furthermore, we have shown that the residual neural network helps to extract deep features while saving computing time *via* processing the convolutional layers in parallel. For feature analysis, the attention-based BiLSTM demonstrated its advantage in addressing problems of long-distance dependency, allowing focus on the most significant features based on the assigned attention values.

## Data Availability Statement

The original contributions presented in the study are included in the article/Supplementary Material, further inquiries can be directed to the corresponding author/s.

## Author Contributions

HZ conceived the study. ZL designed the model and accomplished experiments. ZL and HZ wrote the manuscript.

## Conflict of Interest

The authors declare that the research was conducted in the absence of any commercial or financial relationships that could be construed as a potential conflict of interest.
