# Supplementary material for: Automatic Detection for Multi-Labeled Cardiac Arrhythmia Based on Frame Blocking Preprocessing and Residual Networks
Source: Front Cardiovasc Med. 2021 Mar 19;8:616585. doi: 10.3389/fcvm.2021.616585 (PMC8017170; doi:10.3389/fcvm.2021.616585)

Supplementary Materials

Hyperparameters such as learning rate, regularization, optimization and kernel parameters of hidden layers can be tuned for balancing the memory usage and model performance. This section aims to tune the chosen hyperparameters which involves kernel size and numbers of two types of dense blocks, the number of BiLSTM units, dropout rate and the number of units in the multi-head attention layer. Learning rate is an adjustable parameter in the optimization algorithm, controlling the step of backpropagation and optimization time. The kernel parameters of convolutional layers determine the model performance and the computation load. In the proposed model, dropout layers are added between the average pooling layer, bidirectional LSTM layer and the attention layer. Dropout rate represents the percentage of the activation of neurons which has been deactivated. Shielding neurons properly helps to reduce complex coadaptation between neurons and the number of parameters thereby resolving the problem of over-fitting. In the multi-head attention layer, the input sequences are divided into a given number (number of heads) of segments, and then Attention Mechanism is applied on the corresponding segments. Therefore, the model can attend to relevant information simultaneously from different representation subspace at a different position.

After evaluating the model performance via cross-validation, the most proper setting of hyperparameters is shown in Table S2 and evaluated result is shown in Table S4 in details. The mean squared error on the validation set is used as the standard of evaluation. Procedure of hyperparameter tuning is discussed in the following.

i) Learning rate

This study uses Adam optimizer with a learning rate of 0.001 as suggested (Kingma & Ba, 2014). The decay rate for 1st and 2nd moment estimates was set as default, resulting in fast convergence and less memory requirement.

ii) Kernel parameters of residual neural network

The kernel size and numbers of the convolutional layers are key hyperparameters which should be evaluated. In the proposed model, there are two Conv1D layers in the dense block 1 and three Conv1D layers in the dense block 2. As shown in Supplemental Table 2, the mean square error is lowest when the kernel numbers of the Conv1D layers in the first residual block 1 and 2 are [32,64] and [32,64,64] respectively, meanwhile, kernel numbers of Conv1D layers in the second residual block 1 and 2 are [64,128] and [64,128,128] respectively. Moreover, the optimal kernel size of Conv1D layers in dense block 1 and 2 are [1,1] and [3,3,7] separately.

iii) Units of BiLSTM and multi-head attention

After the proper tuning of the kernel size of the dense blocks, the hidden units of bidirectional LSTM layer, multi-head attention layers, and rate of dropout were set as 128, 256 and 0.5 separately, obtaining the lowest mean squared error.

Table S1. Numbers and distribution of ECG recordings with multiple labels for six different types of abnormalities in CPSC2020.

	AF	I-AVB	LBBB	RBBB	PAC	PVC
AF	0	0	29	172	4	0
I-AVB		0	8	11	4	9
LBBB			0	0	10	0
RBBB				0	57	0
PAC					0	1
PVC						0

Table S2. List of optimal parameters for each layer and residual block in the proposed model.

Layers / Blocks	Kernel number	Kernel size	Pool size
Conv1D	32	3	-
Pooling 1	-	-	3
DenseBlock 1	1st: [32,64]<br>2nd: [64,128]	1,1<br>1,1	-
DenseBlock 2	1st: [32,64,64]<br>2nd: [64,128,128]	3,3,7<br>3,3,7	-
Pooling2	-	-	2
BiLSTM	128	-	-
Attention	256	-	-

Table S3. The optimal hyperparameters of the proposed model.

Hyperparameters	Values
Learning rate	0.001
Dense block 1 kernel number	1st: [32,64]<br>2nd: [64,128]
Dense block 1 kernel size	1,1
Dense block 2 kernel number	1st: [32,64,64]<br>2nd: [64,128,128]
Dense block 2 kernel size	3,3,7
BiLSTM units	128
Dropout rate	0.5
No. of attention units	256
Kernel regularizer. l1_l2	[0.01,0.01]

Table S4. Mean squared error at different sets of hyperparameters.

No. of kernels Residual block 1	No. of Kernels Residual block 2	Kernel size Residual Block1	Kernel size Residual Block2	No. of BiLSTM units	Dropout	No. of Attention units	Mean Squared error
1st: [8,16]<br>2nd: [16,32]	1st: [8,16,16]<br>2nd: [16,32,32]	3,3<br>3,3	5,5,11<br>5,5,11	32	0.3	64	0.050
1st: [16,32]<br>2nd: [32,64]	1st: [16,32,32]<br>2nd: [32,64,64]	3,3<br>3,3	5,5,11<br>5,5,11	64	0.3	128	0.062
1st: [32,64]<br>2nd: [64,128]	1st: [32,64,64]<br>2nd: [64,128,128]	3,3<br>3,3	5,5,11<br>5,5,11	128	0.3	256	0.048
1st: [8,16]<br>2nd: [16,32]	1st: [8,16,16]<br>2nd: [16,32,32]	1,1<br>1,1	3,3,7<br>3,3,7	32	0.5	64	0.063
1st: [16,32]<br>2nd: [32,64]	1st: [16,32,32]<br>2nd: [32,64,64]	1,1<br>1,1	3,3,7<br>3,3,7	64	0.5	128	0.048
**1st: [32,64]**<br>**2nd: [64,128]**	**1st: [32,64,64]**<br>**2nd: [64,128,128]**	**1,1**<br>**1,1**	**3,3,7**<br>**3,3,7**	**128**	**0.5**	**256**	**0.044**

Table S5. Structure and hyperparameters of plain CNN + attention based BiLSTM.

layer	Kernel number/ size
Con1D+BN+RELU	32 / 3
Dropout	0.5
Con1D+BN+RELU	32 / 3
Dropout	0.5
Con1D+BN+RELU	32 / 3
Dropout	0.5
Con1D+BN+RELU	64 / 3
Dropout	0.5
Con1D+BN+RELU	64 / 3
Dropout	0.5
Con1D+BN+RELU	64 / 3
Dropout	0.5
Con1D+BN+RELU	128 / 3
Con1D+BN+RELU	128 / 3
Dropout	0.5
Flatten	-
Bidirectional LSTM	128
Dropout	0.5
Attention	256
Flatten	-
LSTM	256
sigmoid	1

Table S6. Structure and hyperparameters of the challenge-best model in CPSC 2018 (T.-M. Chen et al., 2020).

CNN blocks and layers	CNN layer	Kernel number/size
1	1	32/3
	2	32/3
	3 (pooling)	3
2	4	32/3
	5	32/3
	6 (pooling)	3
3	7	64/3
	8	64/3
	9 (pooling)	3
4	10	64/3
	11	64/3
	12 (pooling)	3
5	13	128/3
	14	128/3
	15 (pooling)	6
Bi-GRU		128
Attention		256
Batch Normalization		
Dense(sigmoid)		

Table S7. Comparison of F1 scores between four different models base on test samples.

Label	Proposed Model F1score	Plain CNN+Attention BiLSTM F1score	Plain CNN+LSTM F1score	Challenge-best F1score
AF	0.959	0.961	0.957	**0.962**
I-AVB	**0.937**	0.878	0.883	0.846
LBBB	**0.958**	0.900	0.792	0.879
Normal	**0.885**	0.817	0.819	0.701
PAC	**0.848**	0.754	0.700	0.649
PVC	**0.920**	0.828	0.838	0.902
RBBB	**0.965**	0.954	0.940	0.904
STD	0.841	0.842	0.852	0.637
STE	**0.868**	0.683	0.667	0.601

Table S8. Compared the F1 scores between the proposed model and the challenge-best model with uses of CPSC2018 and CPSC2020 dataset for various types of abnormalities.

	CPSC2018		CPSC2020	
label	Proposed F1score	Challenge best F1score	Proposed F1score	Challenge best F1score
AF	0.959	0.962	0.940	0.880
I-AVB	0.937	0.846	0.856	0.731
LBBB	0.958	0.879	0.898	**0.355**
Normal	0.885	0.701	0.870	**0.585**
PAC	0.848	0.649	0.743	**0.581**
PVC	0.920	0.902	0.798	**0.247**
RBBB	0.965	0.904	0.922	0.707

Figure S1.Visualization of the ECG lead II waveform of 9 types of cardiac states in CPSC 2018.

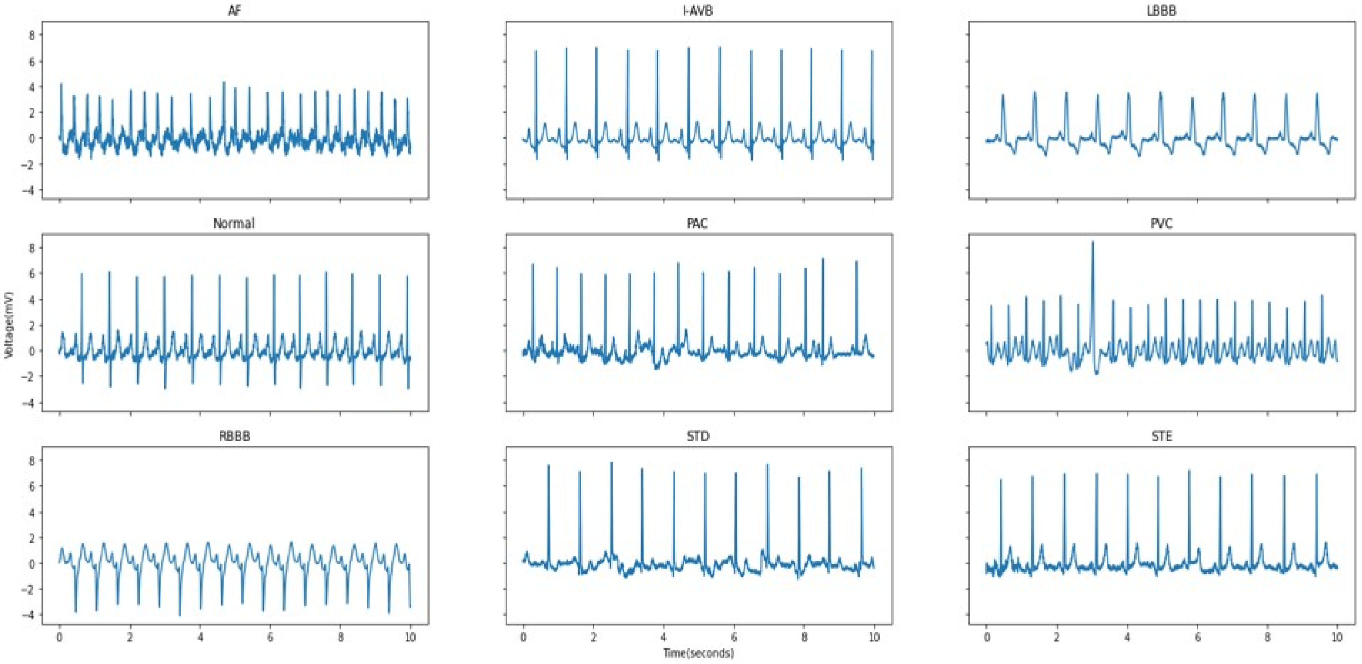

Figure S2. Visualization of the ECG Lead II waveform of a multi-labelled ECG record (A2013).

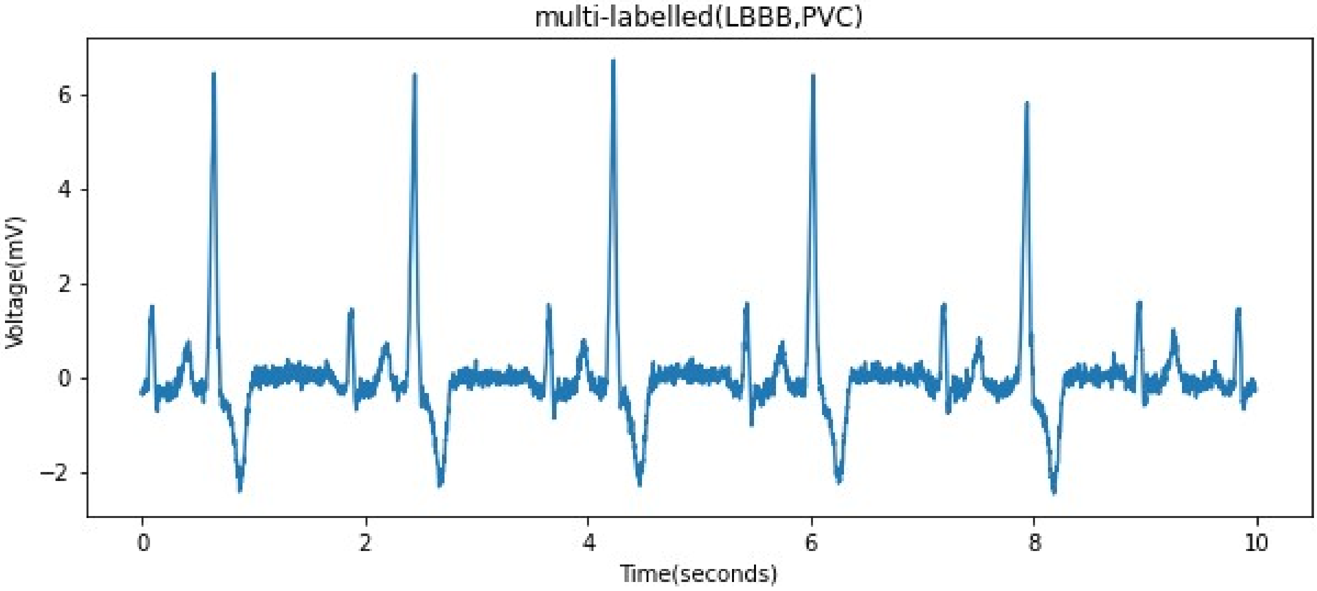

Figure S3. Confusion matrix of 9 types of cardiac states by our proposed model.

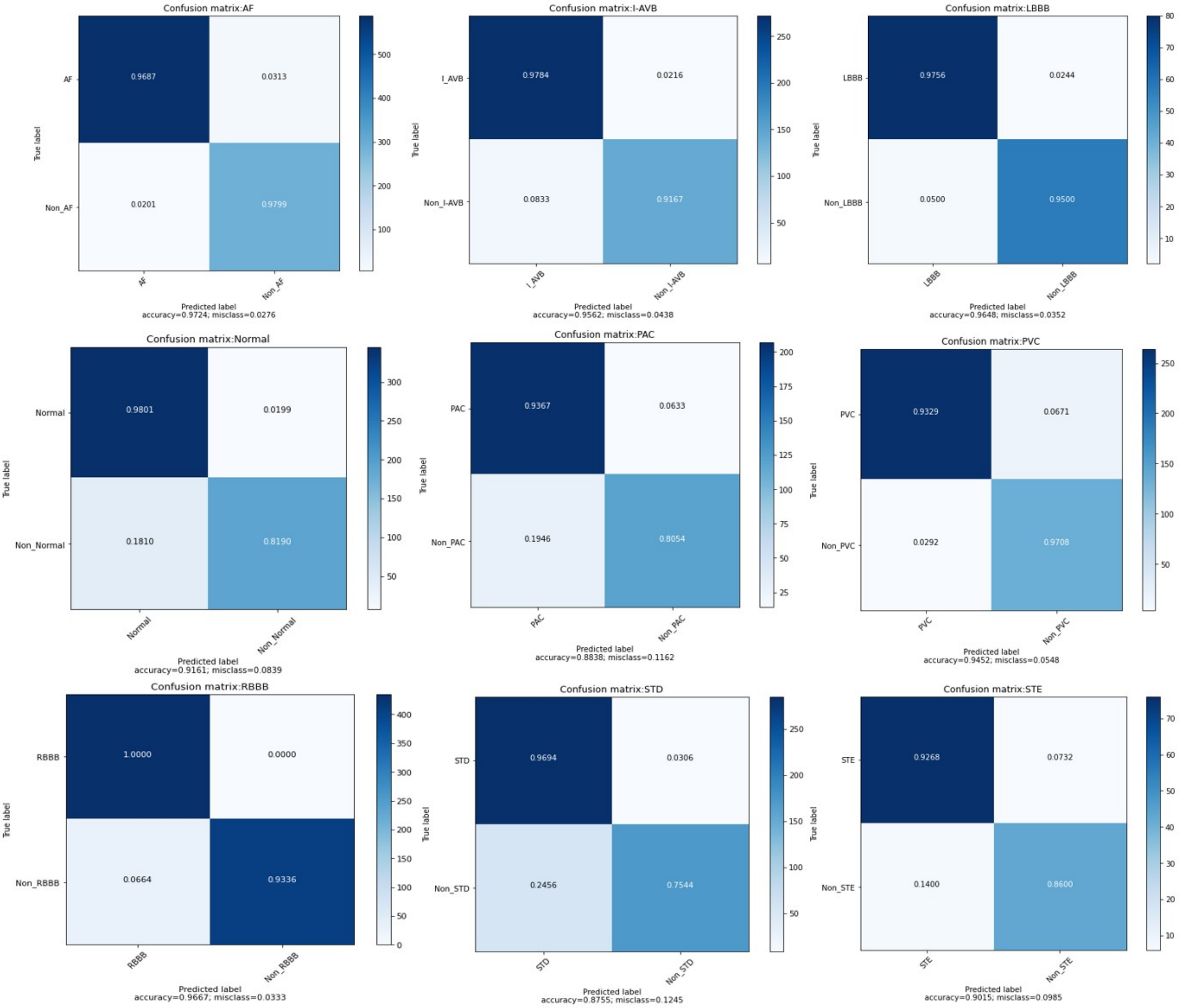

Figure S4. Compared the receiver operator characteristic (ROC) curves for 9 types of cardiac states between 4 different models.

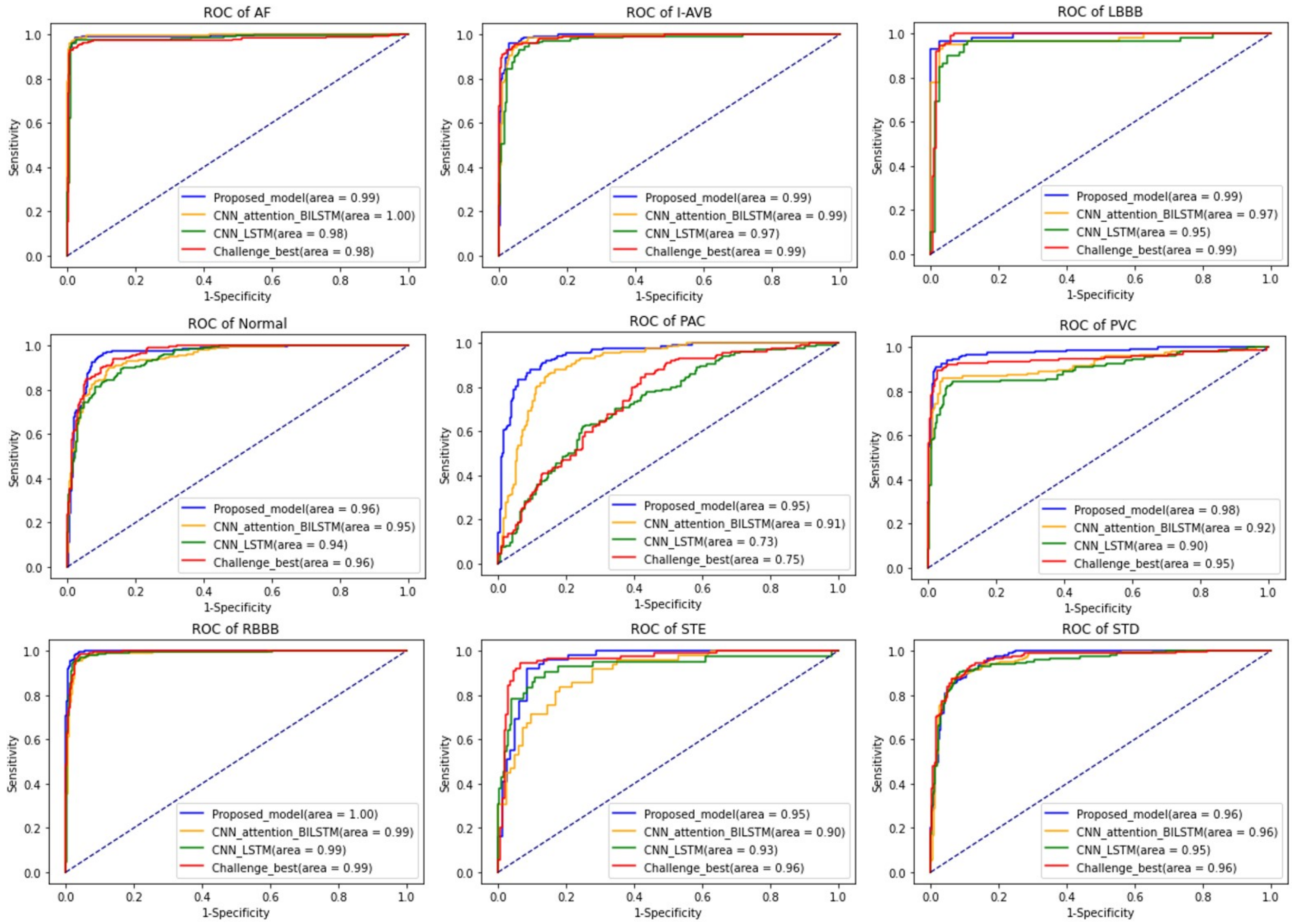

Receiver operator characteristic =ROC, area=area under the curve (AUC)

Figure S5. Compared the Receiver operator characteristic (ROC) curves between the proposed model and challenge best model for 7 types of cardiac states in CPSC 2020.

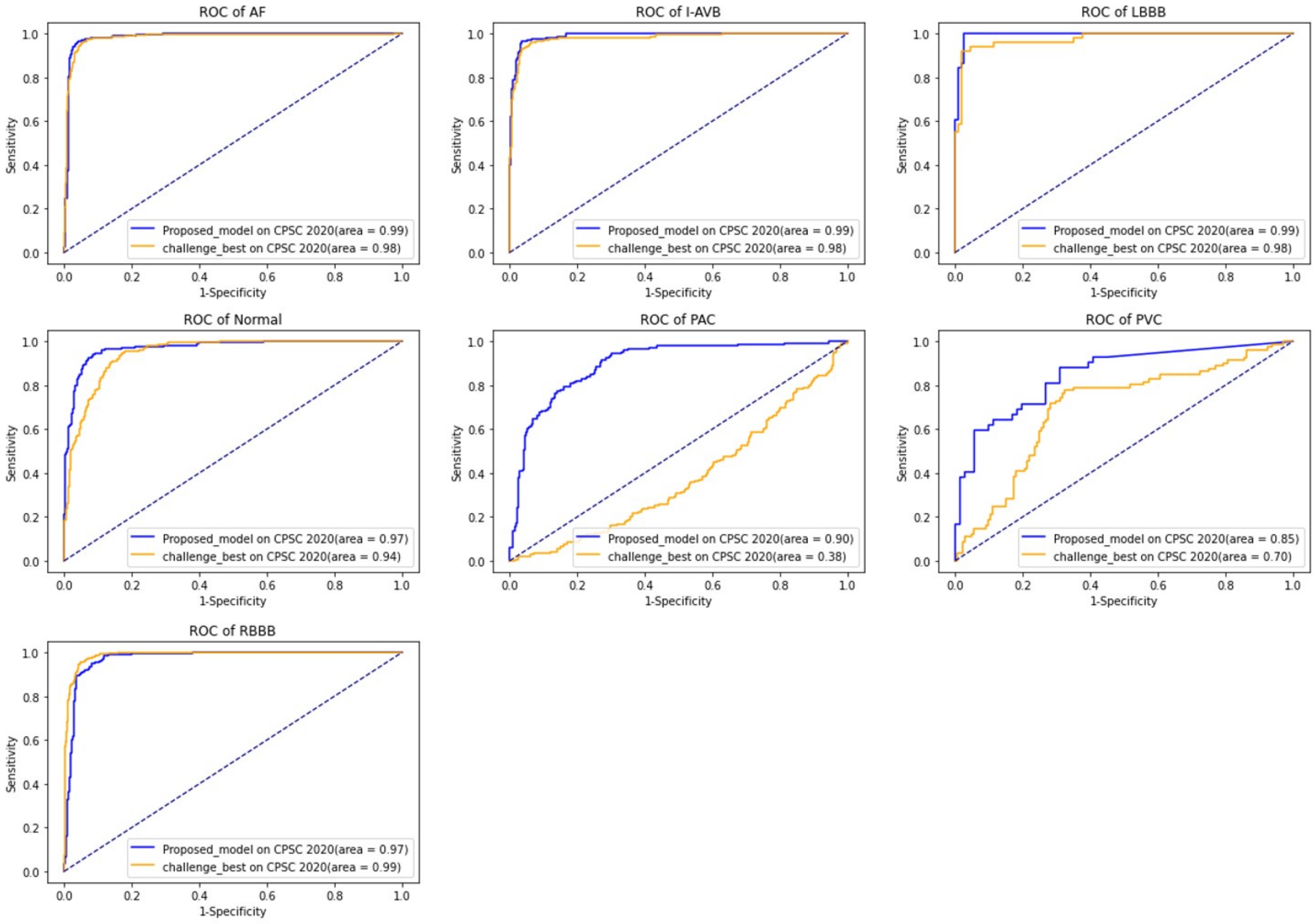

Receiver operator characteristic =ROC, area=area under the curve (AUC)

Figure S6. Receiver operator characteristic (ROC) curves and AUC of 5 diagnosis labels in PTB XL dataset.

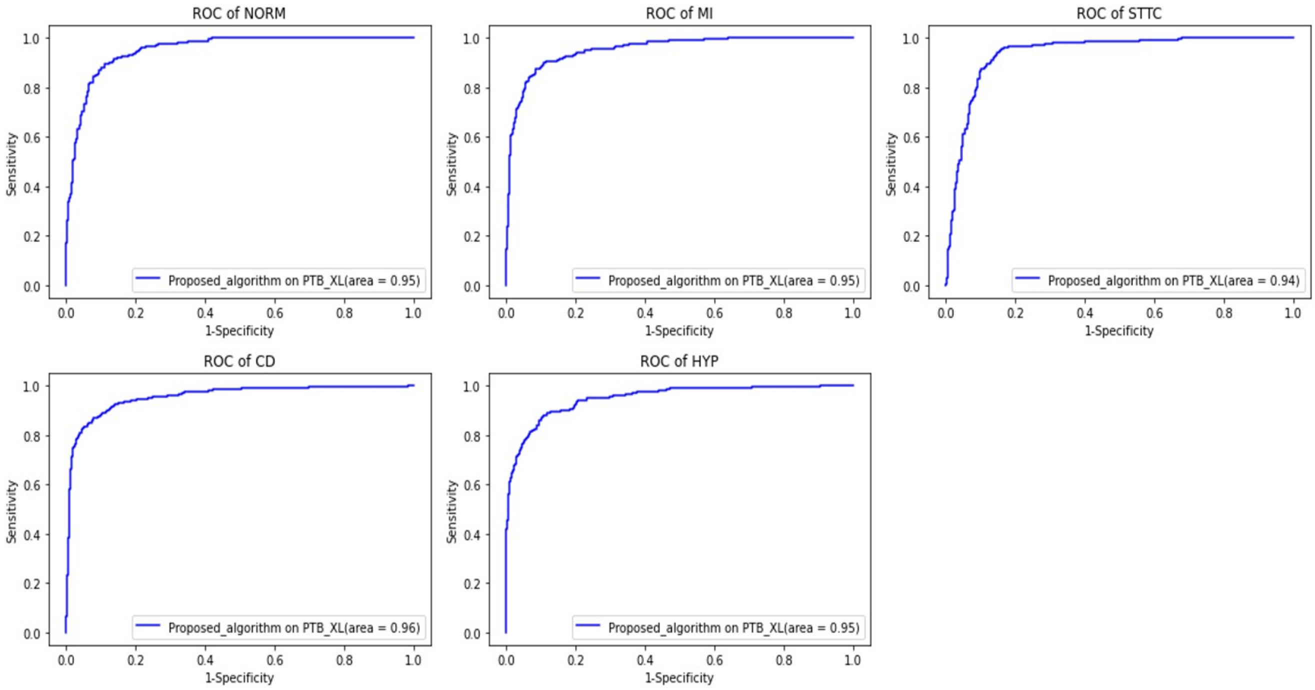

Supplement: Supplementary file 1 [file Data_Sheet_1.PDF]
